# Supplementary figures and images for: Abdominal fat depots associated with insulin resistance and metabolic syndrome risk factors in black African young adults
Source: BMC Public Health. 2015 Oct 5;15:1013. doi: 10.1186/s12889-015-2147-x (PMC4595061; doi:10.1186/s12889-015-2147-x)

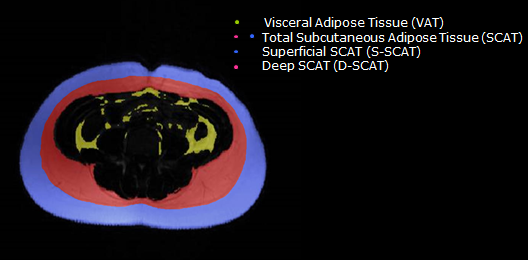

Supplement: Additional file 1: Figure S1. — MRI image taken at the L4 vertebral body, showing visceral adipose tissue (VAT) in green, superficial subcutaneous adipose tissue (S-SCAT) in blue, and deep subcutaneous adipose tissue. (D_SCAT) in pink. (TIFF 429 kb) [file 12889_2015_2147_MOESM1_ESM.tiff]
